# Momentary Environmental Context and Agitation in Persons with Dementia at Home Settings: A Time-Series Analysis

**DOI:** 10.1093/geroni/igaf122.3625

**Published:** 2025-12-31

**Authors:** Chunhong Xiao, Azziza O Bankole, Rita Jablonski, Frank Puga, Arie Nakhmani, Roberto Fernandez-Romero, Tami Wyatt, Robert Davis

**Affiliations:** The University of Tennessee at Knoxville, Knoxville, Tennessee, United States; Virginia Tech, Virginia Tech /Carilion School of Medicine, Virginia, United States; UAB, Birmingham/University of Alabama at Birmingham, Alabama, United States; University of Alabama at Birmingham, Birmingham, Alabama, United States; UAB, Birmingham/University of Alabama at Birmingham, Alabama, United States; The University of Tennessee, Knoxville/University of Tennessee, Tennessee, United States; The University of Tennessee, Knoxville/University of Tennessee, Tennessee, United States; The University of Tennessee, Knoxville/University of Tennessee, Tennessee, United States

## Abstract

Dementia-related agitation often occurs in unpredictable episodes throughout the day, contributing to increased caregiver stress and early nursing home placement. There is a lack of knowledge about the dynamics of momentary agitation influenced by home environment triggers. We conducted a secondary time-series analysis of the Behavioral and Environmental Sensing and Intervention (BESI) dataset to examine how home environmental conditions relate to agitation episodes reported by caregivers of PWD at home. The time-series analysis used data from 13 BESI deployments (Phase II: eight 30-day, Phase III: five 60-day). Environmental features, including light level, temperature, humidity, air pressure, and noise/audio features, were collected via room-level sensors. Agitation was captured using wrist-worn accelerometers. Caregivers provided timestamped agitation reports and contextual surveys. Data were harmonized across modalities using UNIX (a standardized digital timestamp format) and relative time formats. Agitation windows (±5 minutes) were labeled, and environmental features were normalized. Generalized linear mixed models were used to estimate agitation likelihood based on environmental fluctuations, controlling household-level clustering. Preliminary modeling revealed that elevated noise levels and abrupt light changes significantly increased agitation odds (p < 0.05), especially bedroom and kitchen. Caregiver-targeted interventions in Phase III deployments demonstrated reduced agitation sensitivity to environmental volatility. This suggests that real-time contextual feedback enhanced caregiver responsiveness and behavior management. Momentary environmental context plays a critical role in dementia-related agitation. Time-series data offer a novel lens for understanding agitation triggers in dementia care. Findings support the development of context-aware, just-in-time interventions to empower caregivers and improve dementia care in home settings.

